# Development of Physiologically Based Pharmacokinetic/Pharmacodynamic Model for Indomethacin Disposition in Pregnancy

**DOI:** 10.1371/journal.pone.0139762

**Published:** 2015-10-02

**Authors:** Saeed Alqahtani, Amal Kaddoumi

**Affiliations:** Department of Basic Pharmaceutical Sciences, School of Pharmacy, University of Louisiana at Monroe, School of Pharmacy, Monroe, Louisiana, United States of America; Public Health Research Institute at RBHS, UNITED STATES

## Abstract

Findings of a recent clinical study showed indomethacin has lower plasma levels and higher steady-state apparent clearance in pregnant subjects when compared to those in non-pregnant subjects reported in separate studies. Thus, in the current work we developed a pregnancy physiological based pharmacokinetic/pharmacodynamic (PBPK/PD) model for indomethacin to explain the differences in indomethacin pharmacokinetics between pregnancy and non-pregnancy. A whole-body PBPK model with key pregnancy-related physiological changes was developed to characterize indomethacin PK in pregnant women and compare these parameters to those in non-pregnant subjects. Data related to maternal physiological and biological changes were obtained from literature and incorporated into the structural PBPK model that describes non-pregnant PK data. Changes in indomethacin area under the curve (AUC), maximum concentration (C_max_) and average steady-state concentration (C_ave_) in pregnant women were predicted. Model-simulated PK profiles were in agreement with observed data. The predicted mean ratio (non-pregnant:second trimester (T_2_)) of indomethacin C_ave_ was 1.6 compared to the observed value of 1.59. In addition, the predicted steady-state apparent clearance (CL/F_ss_) ratio was almost similar to the observed value (0.46 vs. 0.42). Sensitivity analysis suggested changes in CYP2C9 activity, and to a lesser extent UGT2B7, as the primary factor contributing to differences in indomethacin disposition between pregnancy and non-pregnancy. The developed PBPK model which integrates prior physiological knowledge, *in vitro* and *in vivo* data, allowed the successful prediction of indomethacin disposition during T_2_. Our PBPK/PD model suggested a higher indomethacin dosing requirement during pregnancy.

## Introduction

Indomethacin is a non-steroidal anti-inflammatory drug (NSAIDs) with antipyretic and mild analgesic actions. It has been widely used in the treatment of many arthritic disorders [1, 2]. Indomethacin like other NSAIDs has the ability to inhibit prostaglandin G/H synthase, also called cyclooxygenase (COX)-1 and -2. Prostaglandins formed by COX provoke an inflammatory response. Premature delivery or preterm labor (PTL) is an important obstetrical complication associated with increase perinatal mortality and morbidity. It has been already known that prostaglandins play an important role in the pathogenesis of PTL [3, 4]. Indomethacin has been used for a long time as a tocolytic to treat PTL [3–5].

Orally administered indomethacin is readily absorbed, attaining peak plasma concentrations at about 1–2 hours [6]. The reported bioavailability of indomethacin after oral doses is about 100%, with 90% of the dose absorbed within 4 hours [6]. About 99% of indomethacin is bound to plasma proteins over the expected range of therapeutic plasma concentrations [6]. Indomethacin is metabolized by several drug-metabolizing pathways including those of the cytochrome P450s and UDP-glucuronosyltransferases (UGTs) [7]. *O*-desmethylindomethacin metabolite, which is critical to the elimination of indomethacin, is formed by CYP2C9 [8, 9], while UGT2B7 enzyme plays predominant role in indomethacin glucuronidation in the human liver [10, 11].

During pregnancy, a multitude of temporal physiological, anatomical and metabolic changes take place. Many of these changes may affect the pharmacokinetics and pharmacodynamics of medications. Pharmacokinetic profiles of many drugs are different in pregnant as compared to non-pregnant women mostly due to those changes. During pregnancy, a major hemodynamic changes lead to expansion of plasma volume, increase in cardiac output (up to 50%) and create dilution of plasma proteins, which can alter the apparent volume of distribution (V_d_) of drugs [12, 13]. As a result, concentrations of serum albumin and alpha 1-acid glycoprotein (α1-AGP) decrease by 31% and 19%, respectively, during late pregnancy [12]. Consequently, for some of highly bound drugs, the change of unbound plasma fraction during pregnancy can be dramatic. Enhanced glomerular filtration rate (GFR) during pregnancy leads to increase in the renal excretion of unchanged drugs [12, 13], and changes in hepatic blood flow and hepatic enzyme activity (i.e., intrinsic clearance) have significant effect on drugs hepatic clearance. The hepatic blood flow increases by 60%, whereas, the hepatic enzyme activity increases for some enzymes and decreases for others [14].

Pregnant women and those of reproductive age are usually excluded from clinical studies for ethical and practical considerations. According to the regulations of the Food and Drug Administration (FDA), if pregnancy occurs during a clinical study, the subject is dropped from the study [15]. Consequently, physiologically based pharmacokinetic (PBPK) models that incorporate pregnancy-induced changes in various anatomical, physiological and biological parameters could represent a feasible approach for appropriate dose optimization in pregnant women. These models can be useful to guide dose adjustment in pregnant women to ensure adequate efficacy and to prevent undesirable toxicity. Recently, a number of pregnancy PBPK models have been reported for drugs disposition in pregnant women [16–20]. All these models could successfully explain the altered pharmacokinetics parameters between pregnant and non-pregnant women.

In a recent clinical study, indomethacin pharmacokinetics were determined during pregnancy [21]. Findings showed that indomethacin plasma levels were lower and the steady-state apparent clearance was higher in pregnancy compared to non-pregnant subjects reported in separate studies [21]. The exact mechanism behind this change in plasma levels of indomethacin in pregnant compared to non-pregnant women is still unknown. Therefore, we developed a pregnancy physiological based pharmacokinetic/pharmacodynamic (PBPK/PD) model by considering the essential physiologic and metabolic changes that occur during pregnancy for indomethacin. This model was then used to explain the differences in pharmacokinetic parameters of indomethacin between pregnancy and non-pregnancy. The developed model could be used as a guidance to adjust indomethacin dose during pregnancy.

## Methods

### General workflow of PBPK model development and validation criterion

All PBPK simulations were carried out using the commercially available software GastroPlus^TM^ version 8.5 (Simulation Plus Inc., Lancaster, CA) embedded with the Advanced Compartmental Absorption Transit (ACAT) model and PBPKPlus™ module. Human pharmacokinetics profiles of indomethacin in non-pregnant and pregnant women were taken from literature by scanning with GetData Graph Digitizer version 2.26 and were then used in the modeling [21, 22]. Due to the lack of studies focusing on indomethacin disposition in non-pregnant or pre-pregnant women as well as no studies indicated differences between females and males in indomethacin disposition, the physiology and indomethacin pharmacokinetics in healthy subjects (males and females) available in the literature were used in our modeling strategy as representative of non-pregnant or pre-pregnant women. The general workflow of indomethacin PBPK model development and validation in non-pregnant subjects consisted of the following steps. First, the PBPK model was initially developed using the physicochemical, biopharmaceutical, and pharmacokinetic parameters obtained from literature or estimated by ADMET Predictor (Simulation Plus Inc.) in non-pregnant women population (Table 1), and the model was further validated by comparing the simulated PK data with the observed clinical studies including i.v. dosing, single and multiple oral dosing [22]. Population-dependent physiological parameters in human PBPK models were obtained using the Population Estimates for Age-Related Physiology™ module in GastroPlus. Second, in case that predicted PK profile and parameters were deviant from the observed data the model was refined by parameter optimization by fitting against the non-pregnant clinical data. Third, the pregnancy PBPK model was developed with these verified drug-specific parameters and pregnancy induced physiological changes. The predicted mean values of the PK parameters C_max_ and AUC for non-pregnant and pregnant women as well as these parameters ratios were then obtained based on the simulations.

**Table 1:** Physicochemical and *in vitro* data used in the PBPK model.

Parameter	Value	Methods/references
Molecular weight (g/mol)	357.8	[a]
Log P (at pH 7.4)	4.27	[a]
pKa	4.5	estimated by ADMET Predictor
Solubility at pH 4.2 (mg/ml)	0.04	estimated by ADMET Predictor
Effective permeability (10^−5^ cm/s)	6.17	[b]
*fu*_*p*_	0.01	[a]
Blood: Plasma ratio	0.54	PBPK plus in GastroPlus
V_ss_ (L/kg)	0.23	PBPK plus in GastroPlus
CL_R_ (L/h)	0.96	[c]
CL_H_ (L/h)	7.1	PBPK plus in GastroPlus
PS_TC_ (ml/s)	7.53	estimated by GastroPlus
CYP2C9 V_max_ (μg/min)	2.12	[d]
CYP2C9 K_M_ (mg/L)	1.43	[d]
* *f*_m,CYP2C9_ (%)	55	[d]
UGT2B7 V_max_ (μg/min)	1.05	[e]
UGT2B7 K_M_ (mg/L)	3.57	[e]
*f*_m,UGT2B7_ (%)	33	[e]

[a] drugbank.ca; http://www.drugbank.ca/drugs/DB00328

[b] Caco–2 effective permeability taken from [23].

[c] Renal clearance in humans taken from [8].

[d] Optimized value of *in vitro* data taken from [9] (reported values were 9.9 μM for K_M_ and 0.33 pmol/min/pmol CYP for V_max_).

[e] Optimized value of *in vitro* data taken from [11] (reported values were 17.7 μM for K_M_ and 229.4 pmol/min/mg protein for V_max_).

* *f*_m_ is fraction metabolized

Verification of the established PBPK model was primarily based on AUC and C_max_. Mean AUC, C_max_, and average steady-state concentration (C_ave_) during pregnancy were predicted and compared with published data in pregnant women [21]. The predicted mean population PK parameters of the drug should fall within 80–120% of the observed values (i.e. the predicted/observed ratio should fall within 0.8–1.2).

### Development and validation of PBPK model in non-pregnant subjects

Physicochemical parameters used in non-pregnant PBPK model are summarized in Table 1. Absorption was predicted by the ACAT model, which was used as an input into the PBPK model to predict the plasma and tissue concentration-time profiles after oral administration. The ACAT model was used to quantitatively describe multiple drug absorption contributing factors including drug release, dissolution, precipitation, and passage across the GI membrane. Physiological values for intestinal volumes, transit times, lengths and pH in humans were taken from published data and those built in the software. The contribution of intestinal metabolism was also considered in the simulation by using the *in vitro* K_M_ and V_max_ values for CYP2C9 and UGT2B7 reported in the literature [9, 11]. We assumed rapid partitioning equilibrium of drugs between a homogenous tissue and plasma/blood, and thus perfusion-limited distribution kinetics was used for all tissues (except the liver) in the PBPK model. It has been shown that indomethacin is taken up by primary rat hepatocytes via Na+-dependent and -independent active transport processes [24]. Thus, permeability-limited compartment was used to model hepatic disposition of indomethacin. The following GastroPlus^TM^ built-in equations were used to describe this model:

$$\left({\text{V}}_{\text{e}}+{\text{V}}_{\text{v}}\;{\text{R}}_{\text{bp}}/{\text{K}}_{\text{p}}\right)\;{\text{dC}}_{\text{e}}/\text{dt}=\text{Q}\;\left({\text{C}}_{\text{b}}-{\text{C}}_{\text{e}}\;{\text{R}}_{\text{bp}/}{\text{K}}_{\text{p}}\right)–\left({\text{PS}}_{\text{TC}}\;\left({\text{C}}_{\text{e},\text{u}}–{\text{C}}_{\text{t},\text{u}}\right)+{\text{V}}_{\text{inf}}\;\left({\text{C}}_{\text{e},\text{u}}\right)–{\text{V}}_{\text{eff}}\;\left({\text{C}}_{\text{t},\text{u}}\right)\right),\;\text{and}$$

$${\text{V}}_{\text{t}}\;{\text{dC}}_{\text{t}}/\text{dt}=\left({\text{PS}}_{\text{TC}}\;\left({\text{C}}_{\text{e},\text{u}}–{\text{C}}_{\text{t},\text{u}}\right)\;+{\text{V}}_{\text{inf}}\;\left({\text{C}}_{\text{e},\text{u}}\right)–{\text{V}}_{\text{eff}}\;\left({\text{C}}_{\text{t},\text{u}}\right)\right)–v_{metab}\;\left({\text{C}}_{\text{t},\text{u}}\right)–{\text{Cl}}_{\text{int},\text{u}}\times {\text{C}}_{\text{t},\text{u}}$$

where C_b,_ C_e_, and C_t_ are drug concentrations in blood entering tissue, in the extracellular and intracellular space, respectively (u: unbound); V_v_, V_e_ and V_t_ are the volumes of the vascular, extracellular and intracellular compartments, respectively; K_p_ is the partition coefficient for the extracellular space; R_bp_ is the blood: plasma concentration ratio; PS_TC_ is the permeability and surface area product for tissue; Cl_int,u_ is unbound intrinsic clearance; V_inf_ is the influx transport rate into tissue; and V_eff_ is the efflux transport rate from tissue. *v*_*metab*_ is the metabolism rate in tissue. Tissue-plasma partition coefficients (K_p_) values were calculated using the tissue composition equations according to the relationship between physiological data and compound-specific determinants of distribution like lipophilicity (Log P), ionization (pKa), and plasma protein binding (*fu*_p_) (Table 2) [25]. Kinetic parameters of the uptake of indomethacin by hepatocytes were taken from the literature [24]. Because of the absence of definitive *in vitro* transport studies and presence of multiple uptake mechanisms [24], a lumped transporter was used. PS_TC_ was obtained from *in vitro* data [24], and optimized to fit the plasma profile of indomethacin. The steady state volume of distribution (V_ss_) was then estimated using the following equation:

$${\text{V}}_{\text{ss}}={\text{V}}_{\text{p}}+{\text{V}}_{\text{ery}}\times \text{E}:\text{P}+\sum {\text{V}}_{\text{t}}\times {\text{K}}_{\text{pt}}\times \left(1-{\text{ER}}_{\text{t}}\right)$$

where V_p_, V_ery_, and V_t_ are plasma volume, erythrocyte volume, and tissue volume, respectively; E:P is erythrocyte-to-plasma concentration ratio; K_pt_ is tissue/plasma partition coefficient for tissue t, and ER_t_ is the extraction ratio in the tissue.

**Table 2:** Tissue-to-Plasma Partition Coefficients (K_p_) of indomethacin used in non-pregnant and pregnant (2^nd^ Trimester) subjects PBPK models.

Tissue	Non-pregnancy	Pregnancy
Lung	0.22	0.22
Adipose	0.30	0.30
Muscle	0.07	0.07
Liver	0.08	0.08
Spleen	0.11	0.11
Heart	0.17	0.17
Brain	0.06	0.06
Kidney	0.14	0.13
Skin	0.29	0.28
Red Bone Marrow	0.18	0.17
Yellow Bone Marrow	0.07	0.07
Rest of Body	0.13	0.13
Reproductive organ (fetoplacental unit)	0.15	0.06

Indomethacin is mainly cleared via metabolism by several drug-metabolizing pathways, including those of the CYPs and UGTs. The formation of *O*-desmethylindomethacin by CYP2C9 is critical to the elimination of indomethacin and represents about 55% of total drug elimination in the urine [8, 9], whereas, 33% of total dose is excreted as a glucuronide conjugates by UGT2B7 [10]. Hepatic intrinsic metabolic clearance (CL_int,H_) of indomethacin (28.3 L/h) was predicted using *in vitro* to *in vivo* extrapolation from *in vitro* studies [9, 11].

The initial model was verified by comparing the simulated PK profiles with the observed data for non-pregnant population. If predicted PK profile and parameters were significantly deviant from the observed data, the model would then be refined by parameter optimization by fitting against the non-pregnant clinical data. The GastroPlus optimization module was utilized to estimate parameters that significantly influence PK profiles. Initial scaling using enzymes kinetics values for both enzymes, determined *in vitro*, significantly under-predicted *in vivo* i.v. clearance (CL_IV_) and CL_ORAL_ (S1 Fig). The enzymes kinetics parameters were thus optimized using GastroPlus build-in optimization module^TM^ to capture the PK profile of indomethacin in non-pregnant clinical studies.

### Development and verification of PBPK model in pregnant subjects

**Fig 1:**
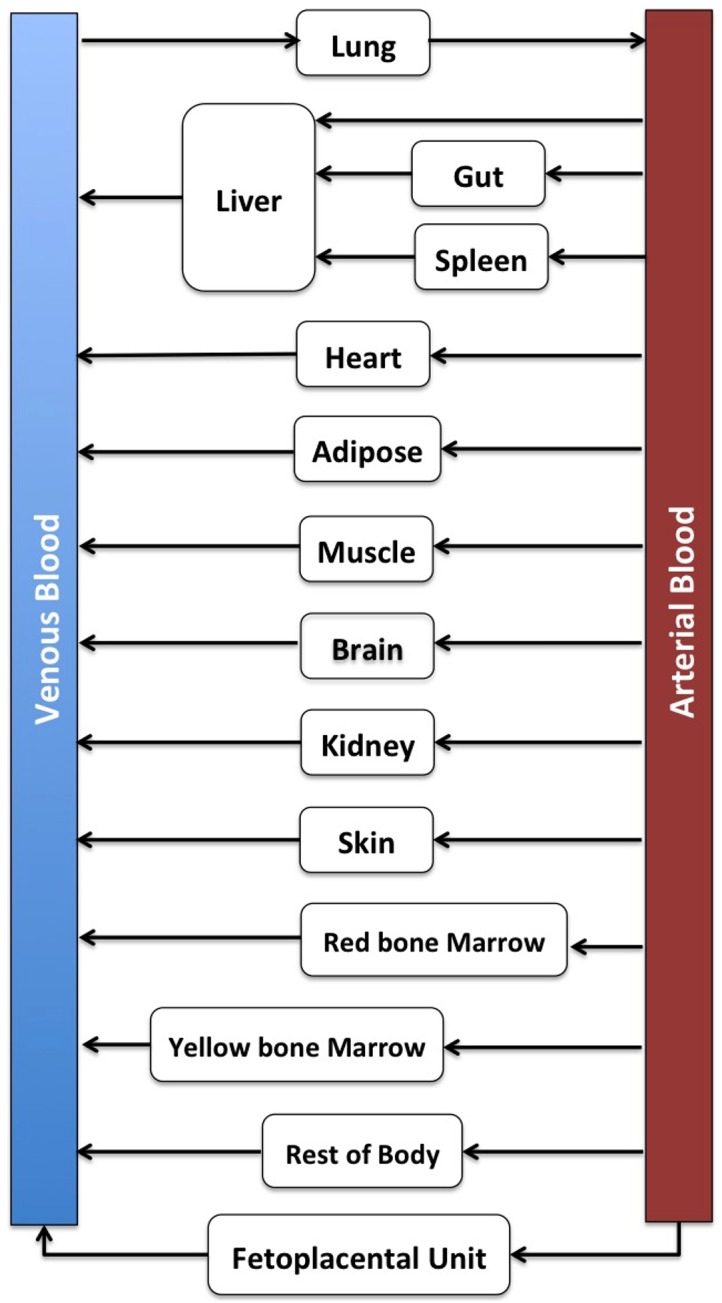
Structure of pregnancy physiologically based pharmacokinetic (p-PBPK) model.

In the present study we simulated the PK of indomethacin in pregnant subjects using PBPK models consisting of 14 compartments representing various tissues (Fig 1). The reproductive organ compartment in the default GastroPlus PBPKPlus module was renamed as fetoplacental unit and used to represent lumped intra-uterine components, including the fetus, placenta, amniotic fluid, and uterus [17]. The compartment of the fetoplacental units was also assumed to be perfusion rate limited. This pregnancy PBPK (p-PBPK) model incorporated the physiological parameters, indomethacin physiochemical properties, ADME data, and information obtained from the clinical studies. The PK data of indomethacin during pregnancy were obtained and digitized from literature [21]. In this clinical study, most of subjects received treatment in the second trimester (mean ≈ 25 weeks; T_2_). The p-PBPK model was developed taking in consideration the physiological changes that occur during pregnancy (e.g., body weight, cardiac output, GFR, CL changes contributed by CYP450 enzymes, volume and perfusion rate). In the model the tubular secretion was held constant due to the low contribution of renal clearance to the total clearance of indomethacin, and the lack of evidence demonstrating changes in tubular secretion in humans during pregnancy. Thus, changes in renal clearance would be mainly affected by changes in GFR and *fu*_*p*_. The volume and perfusion rate for each tissue were automatically calculated using direct model balancing or manually defined using the regression equations reported by Abduljalil et al. [12]. These quantitative regression equations were applied to adjust parameters input (Table 3). Changes in tissues’ volumes and blood flow are presented in S1 Table. Based on the literature, we assumed that the gut physiology is same and that there is no changes in term of absorption between pregnant and non-pregnant subject [12, 17, 26, 27]. The hepatic uptake of indomethacin has been reported to be mediated by different OAT isoforms [24, 28, 29], however due to the lack of reports investigating the effect of pregnancy on the activity of these transporters we assumed the hepatic uptake of indomethacin doesn’t change in pregnancy, and thus kept constant in the p-PBPK model. Various pregnancy-related hemodynamic changes can alter the apparent V_d_ of indomethacin. In this p-PBPK model, the drug distribution during pregnancy was assumed to be mainly affected by changes in body weight, plasma volume and plasma protein levels. There is an average of 16% increase in the body weight during T_2_ of pregnancy and up to 23% increase in late pregnancy [12, 13]. This weight gain during pregnancy is predominantly result from increased total fat mass and body water up to 32% and 41%, respectively. Plasma protein levels generally are decreased during pregnancy relative to pre-pregnancy values, which may alter *fu*_*p*_ of drugs. Serum albumin concentration decreases by 31% during late pregnancy [12, 13]. Additionally, the plasma volume and red blood cell volume may increase up to 50% and 28% during the course of pregnancy, respectively [12, 13]. In the p-PBPK model, this increase in the plasma volume was allocated to the arterial and venous blood pool. The change in the volume of these tissues during pregnancy can be described by using the equations summarized in Table 3. Other physiological parameters that increase significantly during pregnancy are cardiac output and uterine blood flow. The cardiac output increased by 18%, 28%, and 30% in the first, second, and third trimester of pregnancy [13]. Uterine blood flow increases significantly as the uterus receives about 12% of cardiac output at late pregnancy in comparison to 0.5% in non-pregnant women [17]. The drug elimination during pregnancy is significantly changed due to alterations in metabolic enzyme activities, glomerular filtration rate, and renal blood flow rate. The change in hepatic enzymes activity during pregnancy is CYP-isoform specific. Previous studies showed that maternal CYP2C9 activity increased by 40, 50, and 60% during first, second, and third trimester of pregnancy [13, 16]. UGT2B7 enzyme significantly contributes to the metabolism and elimination of indomethacin; and this enzyme has been reported to be induced in pregnancy. Several studies showed that the clearance of different drugs that are mainly eliminated via metabolism by UGT2B7 was significantly higher in pregnant compared to non-pregnant women [30–35]. Among these studies, Gerdin et al reported a 59% increase in the clearance of morphine, a drug that undergoes metabolism by glucuronidation via UGT2B7 enzyme, in pregnant compared to non-pregnant women [30].The predicted mean PK parameters (Cmax, AUC, peak concentration time (tmax), and Cave) for pregnant and non-pregnant women as well as the ratios of the mean PK parameters (pregnant vs. non-pregnant) were then obtained based on the simulations.

**Table 3:** Main anatomical, physiological and biological parameter changes (in 2^nd^ trimester), and regression equations needed for p-PBPK model during different gestation ages (GA) in weeks, based on Abduljalil et al [12].

Parameter (Unit)	Equation	Changes^a^
Total body weight (TBW) (kg)	TBW = 61.1+0.2409 GA +0.0038 GA^2^	↑ 16%
Total fat mass (TFM) (kg)	TFM = 17.14+0.1305 GA+0.0008 GA^2^	↑ 16%
Cardiac output (CO) (L)	CO = 301+5.916 GA−0.088 GA^2^	↑ 27%
Plasma volume (L)	Plasma volume = 2.5−0.0223 GA+0.0042 GA^2^−0.00007 GA^3^	↑ 42%
Red blood cell (RBC) volume (L)	RBC volume = 1.49+0.0098 GA^2^	↑ 20%
Hematocrit (Hct) (%)	Hct = 39.1–0.0544 GA—0.0021 GA^2^	↓ 8%
Albumin (g/L)	Albumin = 45.8–0.1775 GA—0.0033 GA^2^	↓ 16%
Glomerular filtration rate (GFR) (mL/min)	GFR = 114 + 3.2367 GA—0.0572 GA^2^	↑ 37%
Effective renal blood flow (L/h)	Effective renal blood flow = 53 + 2.6616 GA—0.0661 GA^2^	
Uterine blood flow (L/h)	Uterine blood flow = 1.71+0.2068 GA+0.0841 GA^2^−0.0015 GA^3^	↑ 1,567%
Uterus weight (g)*	Weight of the uterus = 80+8.2931 GA+0.3546 GA^2^	508.9
Fetal volume (mL)*	Fetal volume = 0.01 exp(13.604(1−exp(−0.0702GA)))	728
Placenta volume (mL)*	Placenta volume = 0.0−0.0716+0.9146 GA^2^−0.0122 GA^2^	254
Amniotic fluid volume (mL)*	Aminotic fluid volume = 0+1.9648 GA−1.2056 GA^2^+0.2064GA^3^−0.0061 GA^4^+0.00005 GA^5^	705.3
CYP2C9 activity (%)		↑50%^b^
UGT2B7 activity (%)		↑59%^c^

^a^ relative to non-pregnant subjects.

^b^ this value taken from literature [16].

^c^ this value taken from literature [30].

* Fetoplacental volume = Uterus weight + Placenta volume + Fetal volume + Amniotic fluid volume

### Pharmacodynamic model

Indomethacin, a nonspecific prostaglandin synthase or COX inhibitor, has been used to treat PTL effectively for long [36]. COX isoforms, COX–1 and -2, are essential enzymes for converting arachidonic acid to prostaglandins, which regulate cervical ripening, formation of myometrial gap junctions, and uterine contractions [36, 37]. Previous studies have evaluated the relationship between indomethacin dose and effect quantitatively by focusing on a major mechanism of indomethacin: inhibition of the biosynthesis of prostaglandins E_2_ (PGE_2_) [38–40]. In one of these studies, the authors used the PGE_2_ metabolite 13,14-dihydro- 15-keto-PGE_2_ (PGEM) as a surrogate marker instead of measuring PGE_2_, which is unstable in serum, to study the concentration-effect relationships of indomethacin [39]. The mean percent decrease in PGEM plasma concentrations in 8 healthy volunteers at different time points was measured after oral administration of 25 mg of indomethacin [39]. Multiple direct and indirect pharmacodynamic models were fitted to concentration-time and pharmacodynamic effect-time profiles for 25 mg indomethacin to find the best model that describe the relationship between plasma concentration and PD effect of indomethacin.

## Results

### Development and validation of PBPK model in non-pregnant subjects

**Fig 2:**
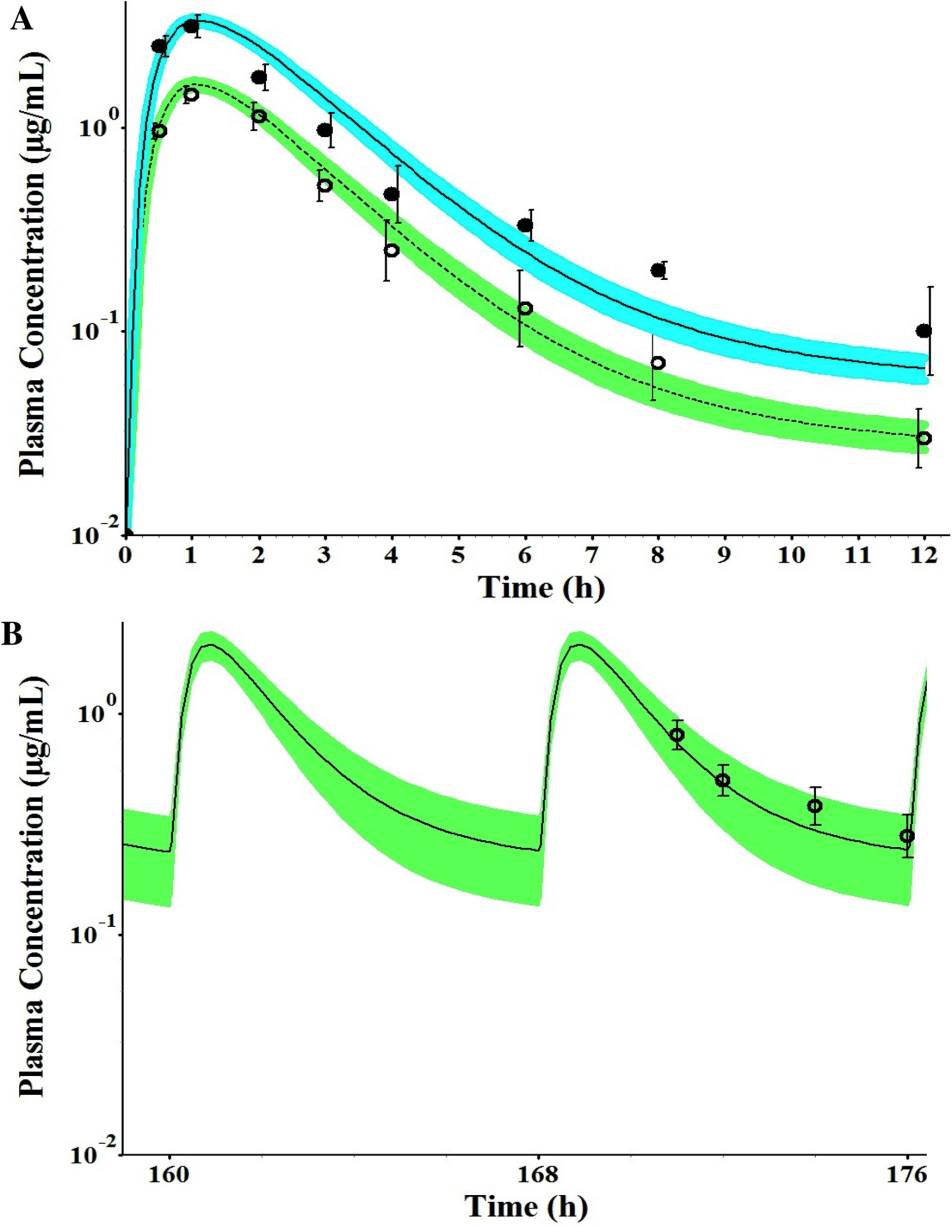
Predicted and observed plasma concentration-time profiles of indomethacin following the administration of a single oral dose of 25 and 50mg. The solid (50 mg) and dashed (25 mg) lines represent predicted mean indomethacin profile (A), and multiple oral dose of 25mg three times daily, the solid line represent predicted mean indomethacin profile (B) to non-pregnant subjects. Mean observed data are overlaid (○ 25mg dose profile; ● 50mg dose profile) [22]. The green and blue shaded areas represent the 90% confidence interval for the simulated data, and error bars represent ±SD.

The PBPK model in non-pregnant subjects was developed using free intrinsic clearance values of CYP2C9 and UGT2B7 enzymes, free fraction in plasma, blood to plasma ratio, permeability in Caco–2 cells, physicochemical parameters (such as molecular weight, pK_a_, and log *P*), and default values of K_p_ implemented in Gastroplus (Table 2). The constructed PBPK model was verified against the disposition kinetics following the administration of a single oral dose of 25 and 50 mg of indomethacin to non-pregnant healthy volunteers [22]. The simulated plasma concentration-time profiles captured the observed PK data (Fig 2A) with regression coefficients (R^2^) greater than 0.95 for both doses. Model-predicted AUC_0-∞_, AUC_0-t_, and *C*_*max*_ all met the verification criterion, with predicted/observed in the range of 0.80–1.2 (Fig 2A and Table 4). At steady-state, following chronic dosing of indomethacin at 25 and 50 mg three times daily, the reported mean AUC_ss_, *C*_*max*_ and *C*_*ave*_ determined by classical compartmental model [2, 22] were also quantitatively predicted, with predicted/observed ratio in the range of 0.80–1.2 (Fig 2B and Table 4). Predicted total systemic clearance of 8.1 L/h was comparable to the average observed values presented in the literature (7.83 L/h) [8, 41]. The predicted V_ss_ for indomethacin was 0.23 L/kg that was comparable to observed values in healthy volunteers studies (0.20 L/kg) [8]. The apparent steady-state oral clearance (CL/F_ss_) was calculated as the dose divided by the AUC_ss_; CL/F_ss_ values obtained from the PBPK model was 5.5 L/h that was comparable to the observed value of 6.1 L/h (Table 4).

**Table 4:** Indomethacin PK parameters after oral dosing in non-pregnant subjects.

	Observed	Predicted	Predicted/Observed
**25 mg** *			
AUC_0-∞_ (μg.h/mL)	4.3±1.1	4.2±1.2	0.98
AUC_0-t_ (μg.h/ml)	4.2±1.3	3.80±1.4	0.92
C_max_ (μg/mL)	1.45±0.4	1.43±0.28	0.98
**50 mg** *			
AUC_0-∞_ (μg.h/mL)	9.4±3.3	9.20±2.75	0.97
AUC_0-t_ (μg.h/mL)	8.69±1.9	8.09±3.2	0.93
C_max_ (μg mL)	3.15±1.8	2.95±0.6	0.93
**25 mg three times daily** *			
AUC_ss_ (μg.h/mL)	4.10±1.5	4.50±1.5	1.09
C_max_ (μg/mL)	1.45±1.1	1.58±0.25	1.08
C_ave_ (μg/mL)	0.51±0.14	0.56±0.23	1.09
Cl/F_ss_ (L/h)	6.10±1.6	5.55±1.25	0.90
**50 mg three times daily** **			
AUC_ss_ (μg.h/mL)	8.56±0.86	9.70±1.3	1.13
C_max_ (μg/mL)	1.9±0.2	2.23±0.53	1.17
C_ss_ (μg/mL)	1.07±0.11	1.21±0.25	1.13

* Reported values in non-pregnant, healthy subjects [22].

**Reported values in non-pregnant subjects [2].

### Development and validation of PBPK model in pregnant subjects

**Fig 3:**
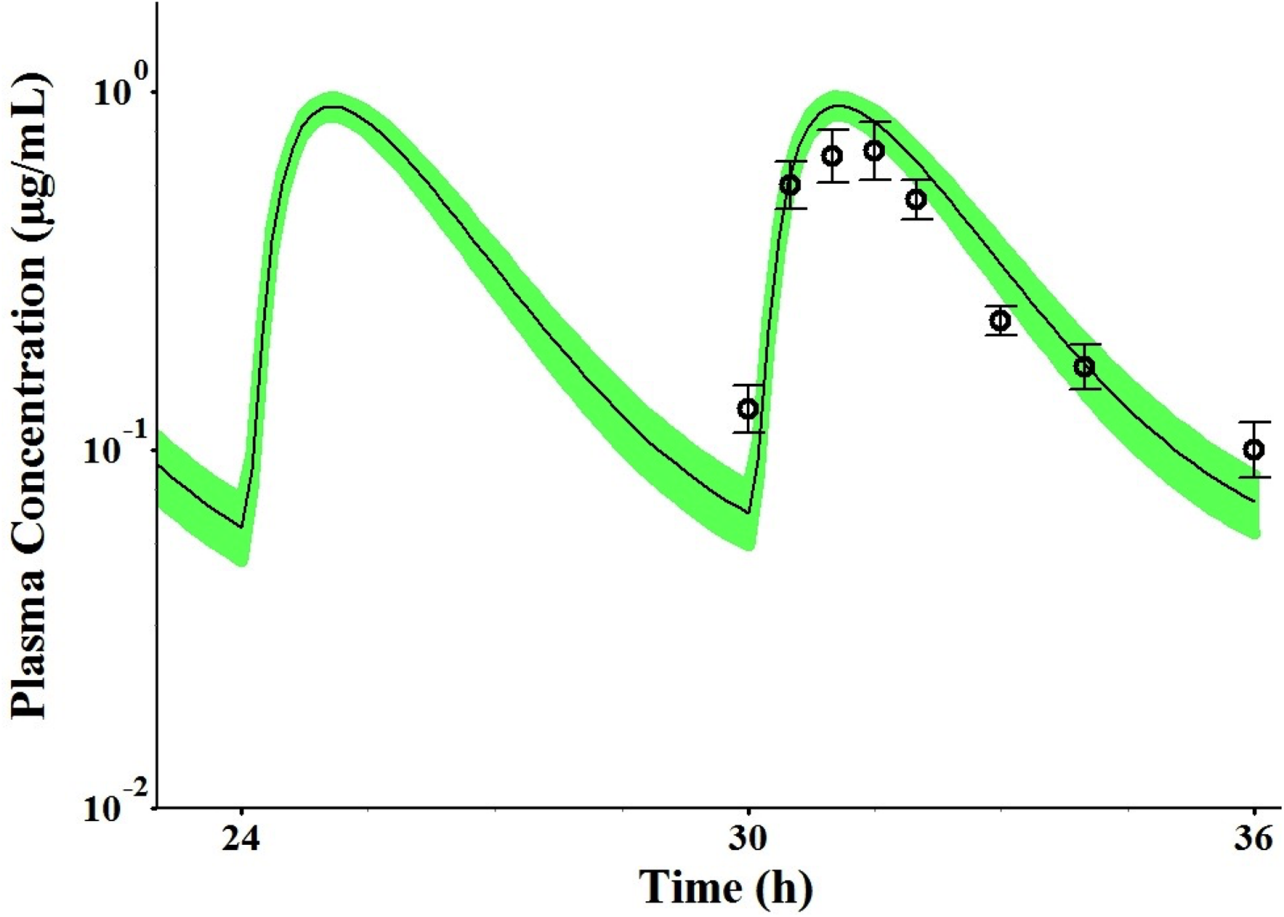
Predicted and observed plasma concentration–time profiles of indomethacin following chronic oral dosing of 25 mg four times daily during the second trimester (T_2_). The solid line represents predicted mean indomethacin profile during T_2_. Mean observed data [21] are represented by the symbol (○). The green shaded area represents the 90% confidence interval for the simulated data, and error bars represent ± SD.

After establishing and verifying the PBPK model in non-pregnant subjects, the pregnancy PBPK model was built by incorporating all physiological changes during pregnancy and addition of the fetoplacental unit compartment. The indomethacin plasma concentration–time profile following a fixed dose of 25 mg four times daily in pregnant women was predicted based on the study design described by Rytting et al [21]. In this study, steady-state indomethacin disposition was evaluated in pregnant subjects during T_2_ of pregnancy. The simulated plasma concentration-time profiles based on the p-PBPK model captured the observed PK data during T_2_ (Fig 3) with R^2^>0.85. Predicted AUC_ss_, *C*_*max*_ and *C*_*ave*_ were comparable with the observed values, with predicted to observed ratios in the range of 0.8–1.2 (Fig 3 and Table 5). The predicted mean ratio of indomethacin (non-pregnant:T_2_) for *C*_*ave*_ was 1.6, compared to observed value of 1.59. The calculated CL/F_ss_ from the predicted values was comparable to the observed value with predicted CL/F_ss_ ratio (non-pregnant: T_2_) of 0.46 that was almost similar to the observed value of 0.42. Indomethacin plasma unbound fraction was predicted to increase from 1% to 1.2% during T_2_. Tissues’ concentrations of indomethacin were also predicted in both pregnant and non-pregnant subjects and are presented in S2 Table. Indomethacin levels in pregnant subjects were lower than those in the non-pregnant as a result of increased clearance.

**Table 5:** Indomethacin observed and predicted PK parameters after oral dosing in non-pregnant and pregnant subjects.

	Pregnant Subjects (T_2_)*			Non-pregnant Subjects**		
PK parameters	Observed	Predicted	Predicted/Observed	Observed	Predicted	Predicted/Observed
AUC_ss_ (μg.h/mL)	1.91±0.53	2.10±0.6	1.09	4.10±1.5	4.50±1.5	1.09
C_max_ (μg/mL)	1.02±0.49	0.98±0.15	0.96	1.45±1.1	1.58±0.25	1.08
C_ave_ (μg/mL)	0.32±0.09	0.35±0.12	1.09	0.51±0.14	0.56±0.23	1.09
CL/F_ss_ (L/h)	14.5±5.5	12.00±2.8	0.83	6.10±1.6	5.55±1.25	0.90

* Reported values in pregnant subjects receiving chronic administration of 25mg of indomethacin four times daily.

** Reported values in non-pregnant, healthy subjects receiving chronic administration of 25mg of indomethacin three times daily.

**Fig 4:**
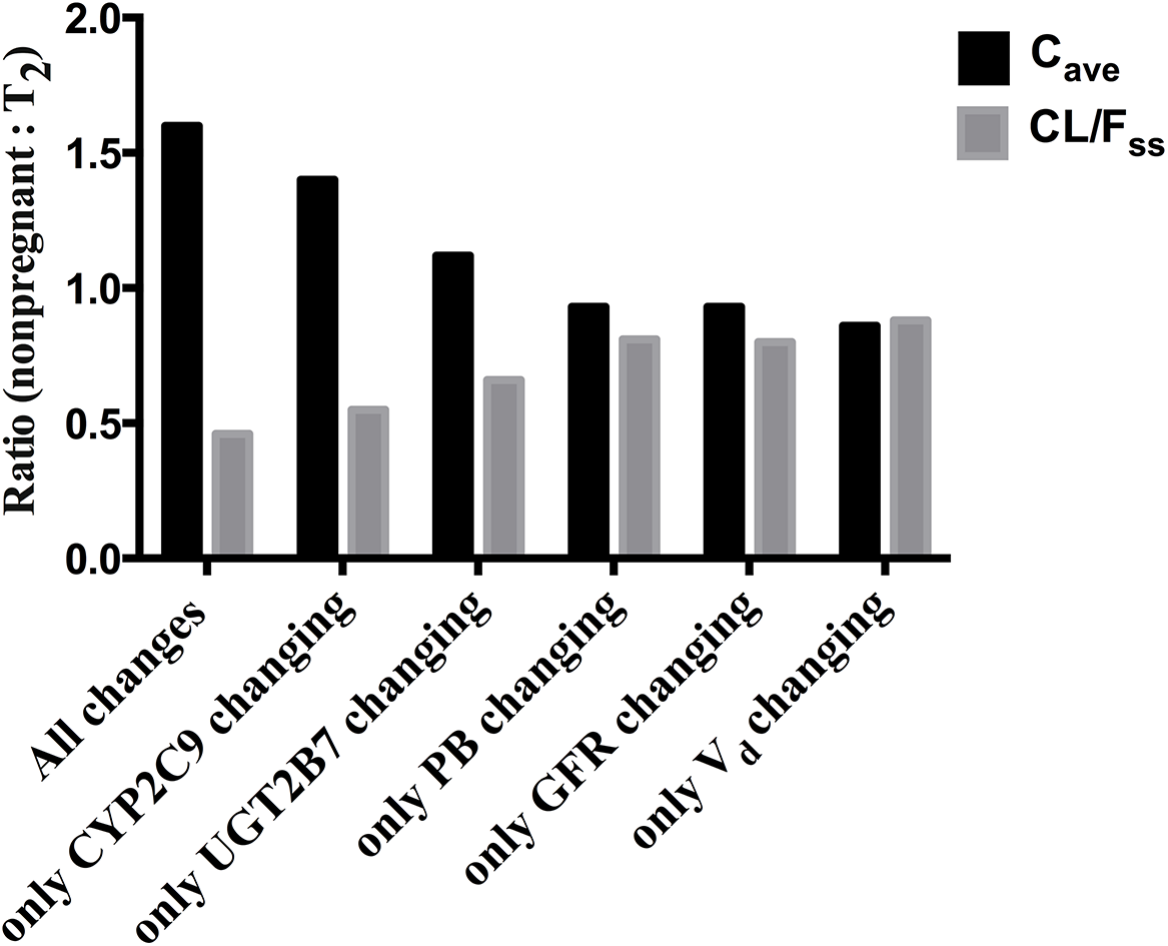
Sensitivity analysis to evaluate mechanism(s) primarily contributes to differences in indomethacin levels in pregnancy. Contribution of changes in metabolism (CYP2C9 and UGT2B7 activities), plasma protein binding (PB), glomular filtration rate (GFR), and volume of distribution (V_d_) to variation in C_ave_ (Black columns) and CL/F_ss_ (Grey columns) during pregnancy.

In addition, a sensitivity analysis was performed to explain the mechanism that primarily contributes to differences in indomethacin plasma levels between pregnant and non-pregnant subjects. The observed increase in apparent clearance and decrease in plasma level of indomethacin during pregnancy could be explained by changes in CYP2C9 activity, UGT2B7 activity, *fu*_p_, GFR, or V_d_. As shown in Fig 4, the sensitivity analysis using our PBPK model indicated that changes (increase) in CYP2C9 enzyme activity as the major contributor to the observed changes in the apparent clearance and plasma levels of indomethacin during pregnancy. The non-pregnant: T_2_ ratios of C_*ave*_ and CL/F_ss_ in only CYP2C9 changing scenario were almost similar to those in the presence of all changes caused by pregnancy (Fig 4). In addition, changing only in UGT2B7 activity contributed to the changes during pregnancy, however to a lesser extent than that observed with CYP2C9. Whereas, changes in protein binding (PB), GFR, and V_d_ showed small impact on the C_*ave*_ and CL/F_ss_ of indomethacin.

### Pharmacodynamic model

**Fig 5:**
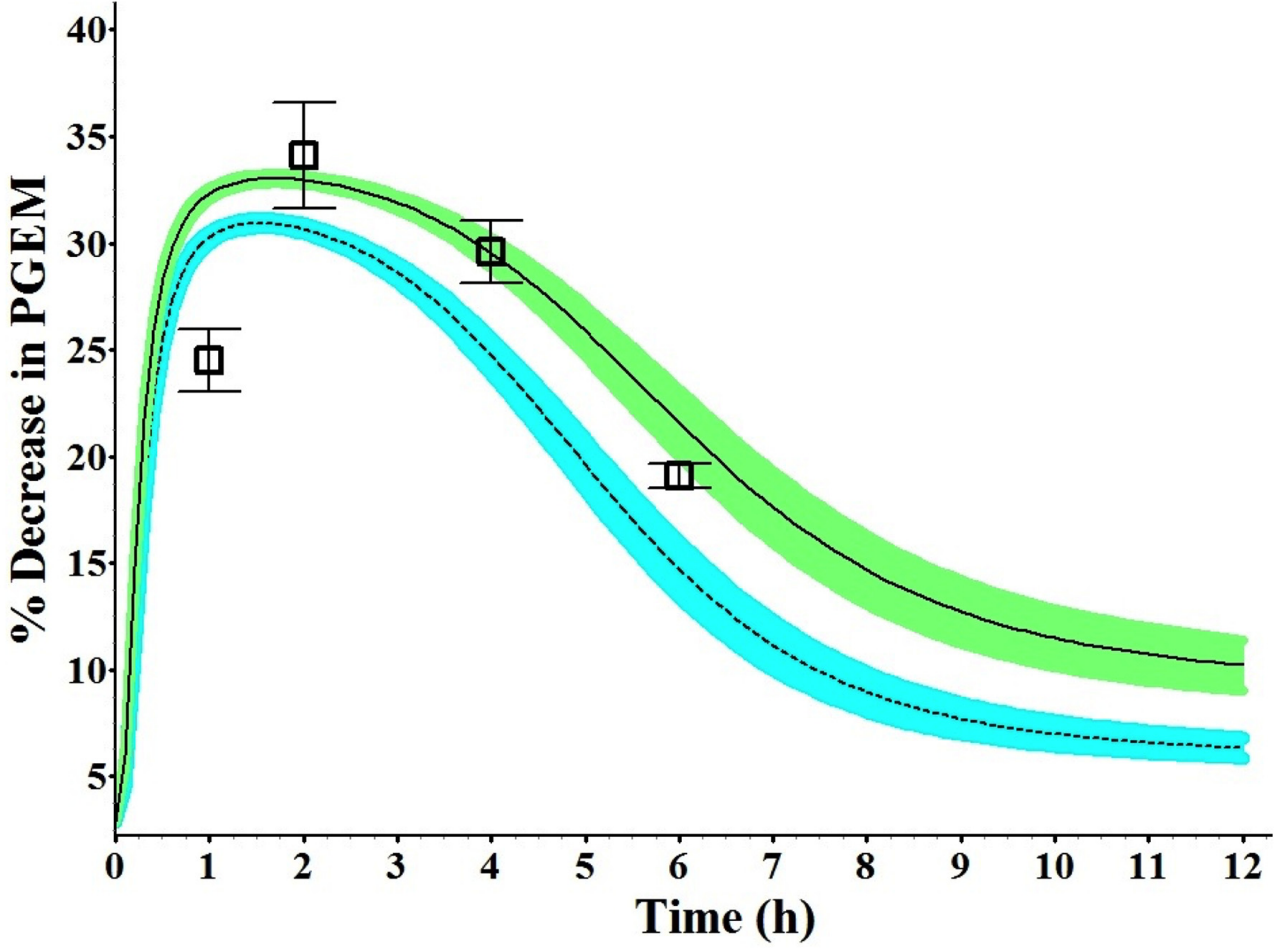
Simulated and observed PD effect-time profiles for indomethacin presented as % decrease in PGEM (13, 14-dihydro-15-ketoprostaglandin E2) plasma concentration vs. time after single oral administration of 25mg. The solid line represents predicted mean indomethacin profile in non-pregnant subjects. The dashed line represents predicted mean indomethacin profile in pregnant subjects. Mean observed data are overlaid for 25 mg dose in non-pregnant subjects [39]. The green and blue shaded areas represent the 90% confidence interval for the simulated data, and error bars represent ± SD.

It has been reported that prostaglandin inhibition, measured by changes in PGEM concentration, correlated significantly with indomethacin concentrations in plasma and could be used to monitor the effect of this drug [39]. Models were fitted for the reported PD response after indomethacin administration based on the drug’s unbound concentration-time profiles. The three direct PD models (Log linear, Emax and Sigmoid) resulted in close matches between observed and simulated effect-time data for indomethacin PD effects measured as % decrease in PGEM plasma concentrations. The three models were used to predict effect-time profile for 25 mg oral dose of indomethacin. The Emax model gave the best prediction of maximum effect for PD effect with prediction errors of less than 5% (Fig 5). The maximum effect prediction error for the Log linear and Sigmoid models was more than 10%. The fitted PD models were then used to predict the effect of 25mg of indomethacin in T_2_ of pregnancy. As shown in Fig 5, 25 mg oral dose of indomethacin in pregnant women in their T_2_ failed to attain the PD effect of indomethacin in non-pregnant subjects indicating the possible need for dose adjustment during pregnancy.

## Discussion

Physiological and biological changes induced during pregnancy can alter the pharmacokinetics and disposition of medications ingested during the term [12, 13]. Pregnant women are actively excluded from the clinical trials for ethical and logistical considerations. Therefore, it’s impossible to study changes in PK of all drugs administered during pregnancy. Since the US FDA encourages drug companies to anticipate and predict drug exposure during pregnancy prospectively, alternative approaches such as mechanistic PBPK modeling to predict drug pharmacokinetics and adjustment of drug dosing regimens are highly desirable. The advantage of such models is their ability to incorporate pregnancy-induced physiological changes, which can serve as a feasible alternative to empirical dose adjustment and guide the clinical trial design in pregnant women.

PTL remains a common complication of pregnancy and causes considerable perinatal morbidity and mortality. Indomethacin is widely used as a tocolytic agent to prevent PTL based on the experience gained from trials. In different placebo-controlled clinical trials, indomethacin has been shown to be superior to placebo in treating PTL [3, 4, 42]. In a recent clinical study, the steady-state pharmacokinetic properties of indomethacin during pregnancy were investigated [21]. The absorption of four daily 25 mg doses of indomethacin at steady-state in pregnant subjects was rapid, with an average t_max_ value of approximately 1 hour that is similar to t_max_ values for indomethacin in non-pregnant subjects [21]. However, the C_*ave*_ value in pregnant subjects who received indomethacin every 6 hours was lower than C_*ave*_ in non-pregnant subjects received the drug every 8 hours. According to Alvan et al study, the C_*ave*_ value following three daily doses of 25 mg indomethacin administered to non-pregnant participants was 37% higher than the C_*ave*_ in pregnant women who received four daily doses of 25 mg indomethacin as reported by Rytting et al [21, 22]. This lower plasma level was related to indomethacin higher clearance during pregnancy [21], where the reported apparent clearance of indomethacin during pregnancy was 14.5 L/h that is greater than the steady-state clearance reported for non-pregnant subjects ranging from 6.0 to 9.8 L/h [21]. The mechanism behind this difference between pregnant and non-pregnant subjects is yet unknown.

In the present study, we developed a PBPK model for indomethacin to explain differences between pregnant and non-pregnant subjects. First, we developed and verified the model in non-pregnant subjects. The PBPK simulations were in agreement with the observed data indicating the model was able to include major processes driving the pharmacokinetics of indomethacin, and have successfully predicted indomethacin pharmacokinetic parameters in non-pregnant subjects (Fig 2 and Table 4). The model predictive performance was reliable as evaluated by predicted AUC and C_max_ values being within ±20% of the observed data. Previous studies demonstrated a rapid elimination of indomethacin, mainly by non-renal routes, and that only 12–14% of indomethacin excreted unchanged in the urine [8–10, 22]. The PBPK model together with the predicted K_p_ values could accurately capture the shape of indomethacin plasma concentration-time profile. As suggested by this model, indomethacin does not distribute widely into tissues due to its high plasma protein binding (99%). The prediction of V_ss_ using this model was within a similar range to the values reported in the literature (0.23 vs. 0.20 L/kg, respectively) [8].

The developed PBPK model, which accounted for metabolizing enzymes induction in addition to other known pregnancy-induced changes including V_d_, *fu*_p_, and CL_r_, has successfully predicted indomethacin disposition during T_2_ and was comparable with the observed data [21]. The observed increases in the apparent clearance of indomethacin during pregnancy may be due to changes in V_d_, urinary excretion, albumin concentrations, or hepatic metabolism of the drug during pregnancy. Changes in drug absorption were excluded because of the complete absorption of indomethacin, suggesting that absorption is less likely to be a significant factor [6, 8, 12, 21, 22]. To determine the major contributing factor to changes in CL/F_ss_ during pregnancy, a sensitivity analysis test was performed by incorporating one change in the PBPK model at a time. These changes include: V_d_, PB, GFR, UGT2B7, or CYP2C9. The significant expansion in the plasma and blood volume during pregnancy and presence of fetoplacental unit are accompanied with changes in the V_d_ of administered drugs. Our PBPK model showed the slight increase in indomethacin V_d_ during T_2_ compared to non-pregnancy (0.27 vs. 0.23 L/kg) has insignificant effect on indomethacin plasma levels or clearance (Fig 4). It is already known that albumin concentration is reduced during pregnancy. Reduced albumin concentrations could lead to an increase in the *fu*_p_ of indomethacin, which may lead to increased drug clearance. The model predicted *fu*_p_ of indomethacin during pregnancy as 0.012, which was higher than that in non-pregnant subjects (0.01), however and as suggested by the sensitivity analysis such increase has no significant effect on indomethacin plasma levels or clearance during pregnancy (Fig 4). Similarly, pregnancy associated increase in GFR showed only a slight increase in indomethacin clearance which doesn’t explain the significantly increased clearance observed in pregnant women. Conversely, our PBPK model suggested that increased activity of CYP2C9 during pregnancy as the major contributor to indomethacin increased clearance; and to a lesser extent by the enhanced activity of UGT2B7 (Fig 4). Indomethacin is extensively metabolized to phase I and phase II metabolites [7, 9–11]. The conversion of indomethacin to O-desmethylindomethacin, which is critical to the elimination of indomethacin, is primarily catalyzed by cytochrome CYP2C9 [9]. CYP2C9 activity increases by 1.5-fold in T_2_ of pregnancy [12, 13]. The underlying mechanism for changes in drugs metabolism during pregnancy remain largely unknown, however, rising concentrations of various hormones (e.g. estradiol) in maternal blood could modulate the activity of metabolizing enzymes [14].

In the absence of human data, findings from animal studies demonstrated prostaglandin levels to decrease in pregnancy as a result of increased metabolism caused by increased levels of the enzyme prostaglandin 15-hydroxydehydrogenase (PGDH), which catalyzes the initial (and probably rate-limiting) step in the metabolic degradation of prostaglandins [43–45]. This increase in PGDH could be related to changes in steroid hormones levels [43–45]. However, available studies have shown that increased production of endogenous prostaglandins is associated with premature labor [46, 47]. Numerous clinical studies have demonstrated indomethacin is very effective in treatment and delaying PTL [3, 4, 48]. The experience of using indomethacin for tocolysis in human pregnancy has been restricted to its prescription at anti-inflammatory doses designed to produce a fast onset of action for a relatively short duration of exposure. Since indomethacin has been very efficient in preventing PTL at the same doses used in non-pregnant subjects, we assumed that the extent of reduction in prostaglandin synthesis for both populations is same. Thus, in the PD model we assumed that the same level of inhibition is desired for therapeutic efficacy. As the therapeutic effect of indomethacin is related to its unbound concentration, lower plasma levels of indomethacin is expected to lower the PD effect and thus fail to achieve the desired pharmacological effect. Findings from the developed PD model demonstrated that 25 mg oral dose of indomethacin during T_2_ of pregnancy failed to attain the PD effect (PEGM inhibition) of the same dose in non-pregnant subjects (Fig 5). In the absence of clinical pharmacodynamics data in pregnant women, results from our PD model suggest that pregnant women may need a higher indomethacin dosage.

Our study provides a good example for the advantage of the application of PBPK modeling during pregnancy as an effective approach to adjust the dose when there are no clinical studies to guide the requirements. However, while the impact and application of PBPK modeling in the process of drugs development has significantly increased, still, it has several limitations that should be recognized to achieve the scope of development and implementation desired. Among these limitations is the imprecise estimation of parameters necessary for accurate prediction which may cause the simulation to fail. For example, a failure to estimate a drug K_p_ value using the *in silico* equations can result in biased prediction of the drug V_d_ [49]. Also, in the absence of *in vivo* distribution data, PBPK models cannot be readily established with high confidence especially in case of pregnancy where multiple physiological and biological changes take place. Additional limitation includes the lack of clinical studies in pre-pregnancy and/or postpartum populations for some drugs. Therefore, as in the case of the current study, available data form clinical studies that include healthy volunteers of males and females are generally used regardless of differences in the pharmacokinetics between both genders. Moreover, a poor characterization of some physiological processes such as enzyme activities, transporters’ abundance, or the absorption process in pregnancy, may cause the model to fail to optimally describe the pharmacokinetic behavior of a drug in this population.

In summary, a PBPK model was developed and successfully predicted changes in indomethacin pharmacokinetic parameters during pregnancy that were in agreement with clinical observations. Our PBPK model was also able to explain the mechanism behind changes in indomethacin plasma levels and clearance observed in pregnant women. The pharmacodynamics model demonstrated a lower PD effect for indomethacin during pregnancy. While the developed model could be used to assist in designing clinical investigations of indomethacin dosage requirements during pregnancy, or possibly applied for drugs with similar clearance pathways to evaluate altered pharmacokinetics in a pregnant population based on healthy volunteers’ data, the results of this PK/PD modelling cannot be directly used in clinical practice without further *in vivo* validation.

## Supporting Information

S1 Fig A. Initial scaling using enzymes kinetics values for CYP2C9 and UGT2B7, determined from *in vitro* studies, significantly over-predicted indomethacin plasma profile as a result of the under-prediction of CLORAL value (2.7 L/h vs. observed value of 7.83 L/h). B. observed and predicted indomethacin plasma profiles after parameters optimization using GastroPlus build-in optimization module. (PDF)

S1 Table Tissues volumes and blood flow in pregnancy and non-pregnancy PBPK models. (PDF)

S2 Table Predicted indomethacin concentrations (μg/mL) in different tissues in pregnant and non-pregnant subjects. (PDF)
