# Supplementary material for: Development of Physiologically Based Pharmacokinetic/Pharmacodynamic Model for Indomethacin Disposition in Pregnancy
Source: PLoS One. 2015 Oct 2;10(10):e0139762. doi: 10.1371/journal.pone.0139762 (PMC4592215; doi:10.1371/journal.pone.0139762)
Supplement: S1 Fig — (PDF) [file pone.0139762.s001.pdf]

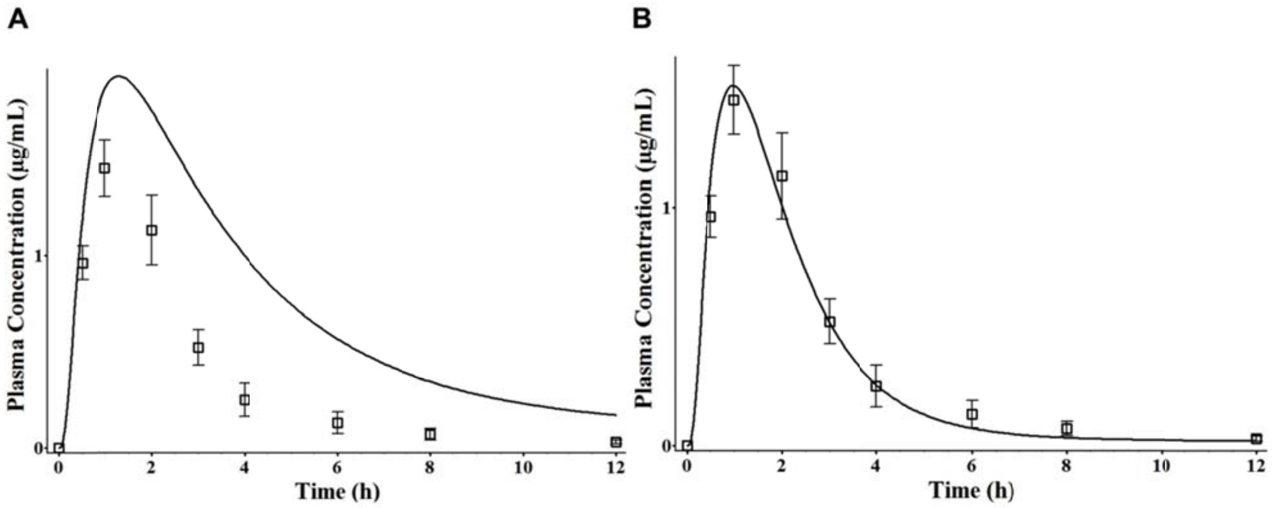

S1 Fig. A. Initial scaling using enzymes kinetics values for CYP2C9 and UGT2B7, determined from *in vitro* studies, significantly over-predicted indomethacin plasma profile as a result of the under-prediction of CL_{ORAL} value (2.7 L/h vs. observed value of 7.83 L/h). B. observed and predicted indomethacin plasma profiles after parameters optimization using GastroPlus build-in optimization module.
