# Supplementary material for: Development of Physiologically Based Pharmacokinetic/Pharmacodynamic Model for Indomethacin Disposition in Pregnancy
Source: PLoS One. 2015 Oct 2;10(10):e0139762. doi: 10.1371/journal.pone.0139762 (PMC4592215; doi:10.1371/journal.pone.0139762)
Supplement: S1 Table — (PDF) [file pone.0139762.s002.pdf]

S1 Table: Tissues volumes and blood flow in pregnancy and non-pregnancy PBPK models.

Tissue	Non-pregnancy			Pregnancy		
	Kp	Volume (mL)	Blood flow (mL/s)	Kp	Volume (mL)	Blood flow (mL/s)
Arterial blood	-	2230.5	101.2	-	2676.55	128.33
Venous blood	-	4460.9	101.2	-	6334.4	128.33
Lung	0.22	1126.9	101.2	0.22	1126.95	128.33
Adipose	0.30	29285.9	9.7	0.30	29285.9	9.7
Muscle	0.07	20984.6	10.5	0.07	20984.6	10.5
Liver	0.08	1651.57	26.3	0.08	1651.57	26.3
Spleen	0.11	175.2	2.92	0.11	175.2	2.92
Heart	0.17	326.9	3.97	0.17	326.9	3.97
Brain	0.06	1739.7	14.78	0.06	1739.7	14.78
Kidney	0.14	285.71	17.52	0.13	285.71	17.52
Skin	0.29	1981.9	3.96	0.28	1981.9	3.96
Red Bone Marrow	0.18	1189.7	5.94	0.17	1189.7	5.94
Yellow Bone Marrow	0.07	3306.91	1.65	0.07	3306.91	1.65
Rest of Body	0.13	13544.2	6.77	0.13	13544.2	8.77
Reproductive organ (fetoplacental unit)	0.15	32.43	0.11	0.06	1719.7	22.32
