# Supplementary material for: Development of Physiologically Based Pharmacokinetic/Pharmacodynamic Model for Indomethacin Disposition in Pregnancy
Source: PLoS One. 2015 Oct 2;10(10):e0139762. doi: 10.1371/journal.pone.0139762 (PMC4592215; doi:10.1371/journal.pone.0139762)
Supplement: S2 Table — (PDF) [file pone.0139762.s003.pdf]

S2 Table: Predicted indomethacin concentrations (μg/mL) in different tissues in pregnant and non-pregnant subjects.

Tissue	Non-pregnancy	Pregnancy
Lung	0.132	0.079
Adipose	1.27	0.45
Muscle	0.027	0.016
Liver	1.01	0.33
Brain	0.047	0.026
Kidney	0.094	0.058
